# Prognostic factors in endovascular treated pelvic haemorrhage after blunt trauma

**DOI:** 10.1186/s12893-017-0283-1

**Published:** 2017-08-09

**Authors:** Rafael Rehwald, Elisabeth Schönherr, Johannes Petersen, Hans-Christian Jeske, Anna Fialkovska, Anna Katharina Luger, Astrid Ellen Grams, Alexander Loizides, Werner Jaschke, Bernhard Glodny

**Affiliations:** 1 Department of Radiology, Medical University Innsbruck, Anichstraße 35, 6020 Innsbruck, Austria; 2 Institute of Neurology, University College London, Queen Square, London, United Kingdom; 3 Department of Trauma Surgery, Medical University Innsbruck, Anichstraße 35, 6020 Innsbruck, Austria; 4 Department of Neuroradiology, Medical University Innsbruck, Anichstraße 35, 6020 Innsbruck, Austria

**Keywords:** Pelvic trauma, Haemorrhage, Transarterial embolization, Endovascular treatment

## Abstract

**Background:**

Angioembolization is the method of choice for treating haemorrhage after blunt pelvic trauma. The aim of this study was to determine technical factors related to endovascular procedures which might be related to patient outcome.

**Methods:**

This retrospective study included 112 consecutive patients (40 women and 72 men; mean age 57.2 ± 20.0).

**Results:**

There were age peaks at 43 and at 77 years. Patients over 65 years had mainly “low-energy” trauma; younger patients were more likely to have polytraumas. Younger patients were more severely injured and had more surgical interventions, larger haematoma volumes, lower Hb levels and required more transfusions than older patients. Women were older than men, had fewer surgeries and waited longer for an angiography (*p* < 0.05 each). Logistic regression analyses identified the injury severity score (ISS) as relevant for survival before age, haematoma volume and Hb. Propensity score analyses showed that in addition to the need for transfusions, haemoglobin, and haematoma volume, the length of the coils and the number of microcoils used were relevant (*p* < 0.05 each). The location of haemorrhage in peripheral parietal arteries (superior and inferior gluteal artery) was an influencing factor for re-angiographies, which were associated with considerably longer hospital stays of more than 40 days. Fewer particles had generally been used in these patients.

**Conclusions:**

The use of too few coils and not using microparticles in angioembolization for pelvic haemorrhage are major influencing factors for the mortality or re-angiography rate. Special attention should be given to thorough peripheral embolization with microcoils, in particular for haemorrhage from the parietal branches of the internal iliac artery.

## Background

Pelvic haemorrhage after blunt pelvic trauma usually occurs in conjunction with severe bone injuries of the pelvis, but can also occur primarily in older people as an isolated result of trauma [1, 2]. In patients over age 65, simple falls at home are the most common cause [1]; in younger people it is more often traffic or sport accidents with large external force [1, 3]. The mortality rate is higher in older patients than in younger ones [3], presumably due to reduced physiological reserves and limited cardiac response to injuries and loss of blood [4]. Risk factors are the severity of the injuries per se, delay in establishing the diagnosis, and insufficient haemostasis [5]. The source of internal haemorrhage, usually located in the pelvic muscles [6] but also in the internal organs protected by the pelvis [5], is 15% arterial and 85% venous or osseous [7, 8]. Arterial bleeding in particular is more important for the prognosis than the bony injury itself [6, 7, 9] and must therefore be treated as quickly as possible [10]. Computed tomography (CT) is the method of choice for a definitive assessment of the type and extent of the injuries [11, 12]. Because CT can also detect arterial bleeding sensitively and specifically, the decision on the further procedure depends largely on the result of the CT scan [9]. For preclinical treatment, the pelvis can be wrapped with blankets or bandages [13]. If haemodynamic stability cannot be achieved through compression [8, 11], further steps to stop the bleeding must be taken [13]. Surgical procedures [14, 15] consisting mainly of the so-called “packing” method where the source of the haemorrhage was packed with towels [15, 16] have been replaced in recent years by arterial angiography with embolization of the injured vessels. The minimally invasive mechanical occlusion of the pelvic arteries makes it possible to quickly and efficiently eliminate various sources of haemorrhage [7, 10, 17, 18], even in haemodynamically unstable patients [15, 19]. The development of adequate material progressed quickly [20, 21], from the initial use of autologous clotted blood and surgical gelatin sponges [22] to modern embolic agents such as coils or polyvinyl alcohol (PVA) particles [20, 21, 23, 24]. However, the significance of the different materials and endovascular techniques with regard to the prognosis of the patient and possible re-intervention is still unclear today. Therefore, we aimed to evaluate the hypothesis that the materials used may impact patient outcome. Accordingly, the primary aim of this study was to assess the use of materials and endovascular techniques with regard to morbidity and mortality.

## Methods

### Type of the study

This study is a retrospective observational study whose implementation had no influence on the treatment of patients. The study complies with the principles of the Declaration of Helsinki in the version of 2013 issued by the World Medical Association.

### Inclusion and exclusion criteria

The study included data sets of patients who had suffered a pelvic trauma in an accident between March 1998 and December 2013 and subsequently had endovascular treatment. Patients whose bleeding was not a result of an accident were excluded from the study. Patients whose radiological images were partially or totally missing or whose images were not usable due to movement artefacts or where the materials used could not be determined were also excluded from the study (*n* = 4).

### Patient management

All patients have been admitted through the accident and emergency (A&E) department and were received by an interdisciplinary team consisting of a trauma surgeon, an anaesthesiologist and a radiologist. Physicians of other specialities, such as neuro-, abdominal- or vascular surgery have been readily available as deemed necessary. An ultrasound system as well as a CT scanner were available directly in the emergency department. After initial patient examination and emergent treatment a CT scan was performed identifying arterial extravasation in all cases included. The trauma team, led by the responsible trauma surgeon, jointly decided on all further steps and made the decision as whether to perform immediate surgery or to refer the patient to the interventional radiologists on duty, who were ready for intervention within 30 minutes in all cases. All patients were treated following the same procedures without exemption.

### Methodology of the study

Using the radiology information system (RIS), 112 patients were identified who had undergone angioembolization of the pelvic vessels due to haemorrhage after a blunt trauma during the period specified. Over the entire period assessed in this study no patients with traumatic arterial haemorrhage were identified who did not receive angio-embolization and no surgical packing procedures were performed during the observed period. The radiological examinations of these patients were assessed using a Picture Acquisition and Communication Software (IMPAX EE20 XII SU1, Agfa HealthCare NV, Mortsel, Belgium) by consensus of three radiologists (R.R., J.P. and B.G.) and a specialist in traumatology (HC. J.). The volumes of the pelvic haematoma were measured using pre-interventional CT scans on a 3D workstation (AW 4.6, VolumeShare 4.4, General Electric Company, Fairfield, Connecticut, USA). The haematoma were defined manually and then automatically segmented. All collected data were anonymised and documented using the Excel software (Microsoft Corp., Seattle, Washington, USA).

### Technical equipment and materials

The pre-interventional CT scans were made – with a few exceptions – on three devices by General Electric (LightSpeed Qxi; VCT; Discovery CT 750 HD; General Electric Company, Fairfield, Connecticut, USA). Each protocol included a late arterial image of the pelvis after the intravenous administration of the contrast medium (Jopamiro® 370; Bracco Imaging S.p.A., Milan, Italy, or Ultravist® 370; Bayer Schering Pharma AG, Berlin, Germany). The dosage in ml corresponded to 1.5 times the estimated body weight in kg. In most cases, an automated dosage modulation program was used, which regulated the tube current at a fixed tube voltage of usually 120 kV, so that a predetermined noise factor of 21 was not reached. Various angiography equipment of the companies Siemens and Philips were used (Siemens Artis zee, Siemens Healthcare GmbH, Erlangen, Germany; Philips Integris H5000, Philips Allura Xper FD20/20, Philips Allura Xper FD20; Koninklijke Philips N.V., Eindhoven, The Netherlands). The angiography and embolization materials used are listed in Table 1.

**Table 1:** Materials used for angiography and TAE

Embolic agents	Manufacturer	Product	Structure	Size	*n*
Coils	Boston Scientific Corp.	IDC™ Interlocking Detachable Coils	nf	Micro	40
		Interlock™ - 18 Fibered IDC™ Occlusion System	f	Micro	4
		Interlock™ Coils	f	Micro	65
		Interlock™ - 35 Fibered IDC™ Occlusion System	f	Macro	4
	COOK® Medical	Detach-11™ Detach Embolization Coil System	nf	Micro	98
		Detach-18® Detach Embolization Coil System	nf	Micro	12
		“MWCE” Embolization Coil	f	both	3^a^/29^b^
		ABC Embolization Microcoils™	f	Micro	7
		MReye® Embolization Coils (“0.035”)	f	Macro	174
		Hilal Embolization Microcoils™	f	Micro	154
	MicroVention, Inc.	MicroPlex® Coil System	nf	Micro	104
	MicroTherapeutics, Inc.	“KA” family	nf	Micro	4
		“KB” family	nf	Micro	1
		“KD” family	nf	Micro	41
		“J” family	nf	Micro	11
		Topaz family	nf	Micro	12
		Dendron family	nf	Micro	6
	*Not identified coils*	-	-	-	99
Particles	Boston Scientific Corp.	Contour™ PVA particles	-	-	52
	Nycomed Amersham plc	Ultra Drivalon PVA particles	-	-	5
	BTG plc	BeadBlock® Embolic Device	-	-	1
	*Not identified particles*	-	-	-	4
Acrylic Glues	Ethicon, Inc.	Ethibloc®	-	-	16
	GEM s.r.l	Lipiodol-Glubran 2®	-	-	1
	*Not identified*	Lipiodol (only)	-	-	3
Other					
	Boston Scientific Corp.	FastTracker™ Microcatheter family			
		Fathom™-16			
		Mustang™ Over-The-Wire Balloon Dilatation Catheter			
		Renegade™ HI-FLO™ Microcatheter			
		Renegade™ STC 18 Microcatheter			
	Medi-Tech, Inc.	Occlusion Balloon Catheter			
	Target Therapeutics, Inc.	Coil Pusher-10			
		Coil Pusher-14			
		Coil Pusher-16			
		Coil Pusher-18			
		TurboTracker-18 Infusion Catheter			
	Codman & Shurtleff, Inc.	Prowler® Select® LP ES			
	Cordis®	STABILIZER® Balance Performance Steerable Guidwire			
	COOK® Medical	D.A.S.H.® Extractor Catheter			
		Flexor® Introducer with small Check-Flo® Valve			
		MicroFerret® - 18 Zeta Infusion Catheter			
	ev3™ Inc.	Silverspeed™ Hydrophilic Guidewire “SilverSpeed-14”			
		X-Pedion™ Hydrophilic Guidewire “X-Pedion 14”			
	St. Jude Medical™, Inc.	AMPLATZER Vascular Plug II			

*TAE* Tansarterial embolization

^a^Microcoils

^b^Macrocoils

### Parameters measured

From the CT data sets, all injuries in different parts of the body were identified and classified using the Abbreviated Injury Scale (AIS), from which the Injury Severity Score (ISS) was then calculated. The type of haemorrhage was assessed and the source of the haemorrhage was mapped anatomically using the CT scan and the angiographic images. If several branches of a vessel e.g., the pudendal artery or the obturator artery were injured, they were assigned to the common main stem and counted as one vessel. The cause of the accident and the time of the accident, of the initial CT scan, of establishing the diagnosis and of the angiographies were recorded. Moreover, data regarding lethality, amount of blood transfusions, length of intensive care stay, clotting factors and stabilization techniques were collected. The additional parameters are listed in Table 2.

**Table 2:** Other parameters assessed

Admission	*n*	Injured Vessel	*n*	Cause of Accident	*n*	OTA	*n*
Emergency department	53	Internal pudendal artery	29	Hit by vehicle	21	A	16
Secondary	48	Obturator artery	29	Hit by heavy object	15	B	46
Casualty department	11	Superior gluteal artery	28	Fall from standing height	15	C	40
		Internal iliac artery	24	Winter sport accident	14	Bruise	10
Discharge	*n*	Pudendal artery	21				
Home	29	Inferior gluteal artery	15				
Rehabilitation	33	Inferior epigastric artery	11				
ADL. external Hospitalization	33	Circumflex femoral artery	10				
		Corona mortis^a^	7				
		Lubar arteries	6				
		Iliolumbar artery	5				
		Lateral sacral artery	5				
		Vesical arteries	5				
		Superficial femoral artery	4				
		External iliac artery	2				
		Gluteal artery	2				

*ADL* Additional

^a^Anastomosis between inferior epigastric artery and obturator artery

### Statistics

Descriptive statistics were generated using the Excel spreadsheet software (Microsoft Corp., Seattle, Washington, USA). Binary and nominal or ordinal categorical codes were introduced as needed. Univariate analyses were performed using the GraphPad PRISM 6 statistical software (GraphPad Software Inc., La Jolla, California, USA). The Fisher-Yates test or the chi-square test was used as appropriate to analyze categorical variables. Distribution analyses were performed with the D’Agostino-Pearson test and two groups were compared using the non-parametric Mann-Whitney test. A *p* < 0.05 was considered significant in each case. The effects of different parameters on dichotomous variables were determined using binary logistic regression analyses. The hypothesis-guided selected variables were tested using the enter method (SPSS, IBM Inc., Chicago, Illinois, USA) and evaluated based on the Wald criterion, the odds ratio, and their significance. On this basis, forward analyses were carried out and their quality was assessed on the basis of the omnibus test of model coefficients and their variance explained using the Nagelkerke pseudo-coefficient of determination. The effects of different parameters on continuous variables were determined using linear regression analyses. Potential predictors were tested using the inclusion method and evaluated using the regression coefficient B, its standardized β, and the t-test.

Then propensity score matching [25] was conducted for the variables mortality, re-angiography, and surgical procedures before angiography using the method proposed by Iacus et al. [26] The largest reductions of the multivariate imbalance measure L1 [26] were achieved using nearest-neighbour matching with a random matching order and a logit estimation algorithm. The target matching ratio was 1:2, with a caliper of 0.2. The replacing of matches algorithm was allowed. No case remained unmatched and no covariables remained unbalanced. A matching ratio of 1:1.93 was achieved for mortality and a ratio of 1:2 for the other two variables. Variables included for adjusting were: 1) surviving vs. deceased patients: patient age, gender, cause of accident, direct or secondary admission, OTA classification, which arteries were injured and number of arteries injured; 2) re-angiography: patient age, gender, cause of accident and direct or secondary admission; 3) surgery before angiography: patient age and gender.

The groups that were formed were then compared using Fisher’s exact test or the Mann-Whitney test. A *p* < 0.05 was considered to be statistically significant.

## Results

**Fig. 1:**
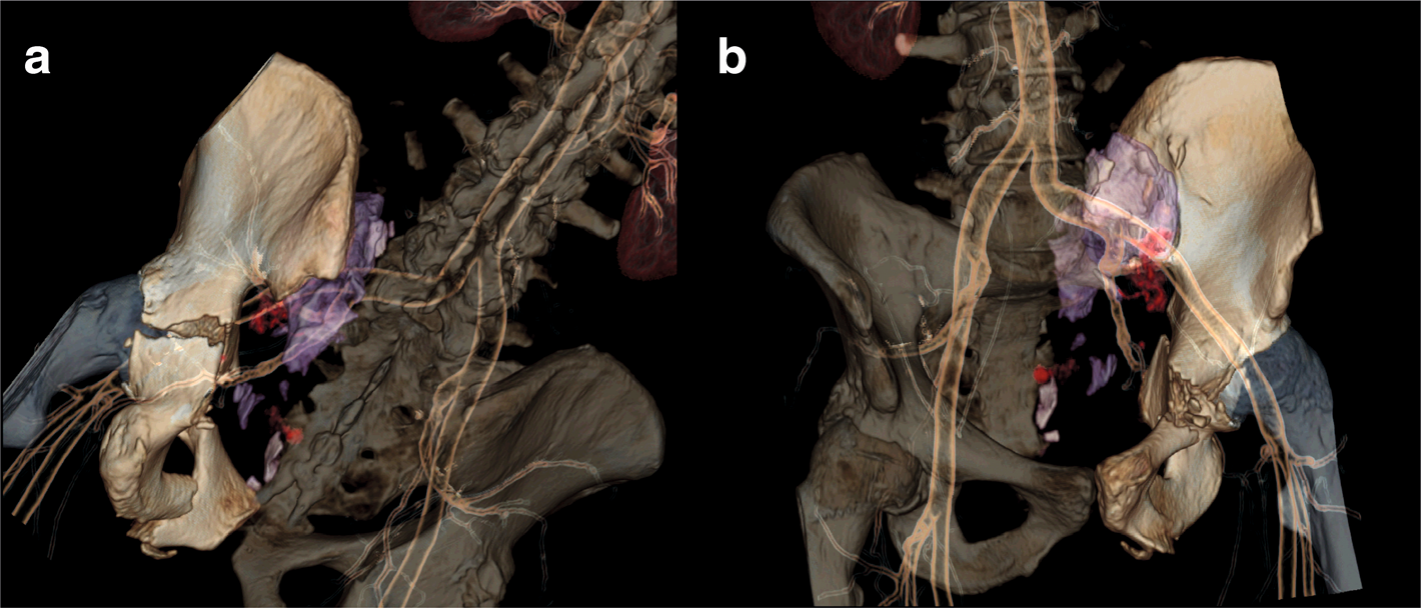
**a** and **b**: *3D Volume rendering*. 3D volume rendering reconstructions of the initial CT scan prior to endovascular intervention in an anterior (**a**) and a posterior (**b**) view. Central acetabular dislocation fracture on the left side (OTA 62.B1.1), and unstable pelvic fracture (OTA 63.C1.3) with vertical sacral fracture and anterior pelvic ring disruption. Disruptions of the left superior gluteal and a sacral arteries with large extravasation of contrast material

The age of patients was 57.2 ± 20.0 years at the time of the accident. Two age peaks were differentiated at 44 years and at about 77 years, with a minimum at about 65 years. The causes of accidents and the type and severity of the pelvic injuries are described along with the other parameters in Table 2. Figure 1a and b show 3D volume rendering reconstructions of the computed tomography of a patient suffering from severe haemorrhage from the left internal iliac territory and pelvic injuries due to a skiing accident.

### Patient characteristics

All patients have been referred to interventional treatment at least once, 17 patients twice and one patient three times. No surgical attempts were made for hemostasis, neither was pelvic packing performed. Surgery before angiography mainly consisted of osteosynthesis procedures and installation of one or more Fixateur externe, either in the pelvis, the extremities or both. Within the observed study group 17 patients did not survive their injuries. In three cases (2.7%) the cause of death were major head injuries, in four cases (3.6%) multi-organ failure and in 10 cases (8.9%) exsanguination. The detailed causes of injury, amount of blood transfusions, clotting factors, and other parameters describing the conditions of the patients are shown in Tables 2 and 3.

**Table 3:** Comparison of patient groups

	First group		Second group		*p* value
	${\overset{-}{x}}_1\pm {\sigma}_1$	*n*_1_	${\overset{-}{x}}_2\pm {\sigma}_2$	*n*_2_	
Patients under 65 vs. over 65 years					
ISS	27.9 ± 14.3	66	17.1 ± 13.3	46	< 0.0001
Surgical procedures	2.2 ± 1.4	66	1.1 ± 1.1	46	0.0001
Transfusion received	44 yes / 22 no	66	18 yes / 28 no	46	0.0065
Hb (g/l)	89.6 ± 28.8	66	100.8 ± 23.6	46	0.0115
Haematoma volume (ml)	789.9 ± 484.0	66	614.0 ± 400.3	46	0.0325
Hospitalization (days)	34.6 ± 27.4	66	25.5 ± 27.1	46	0.0376
Survival	8 yes / 58 no	66	9 yes / 37 no	46	0.2974
Male vs. Female patients					
Surgical procedures	1.9 ± 1.4	72	1.4 ± 1.5	40	0.0393
Duration trauma-angiography (min)	393.7 ± 1088.0	69	405.5 ± 463.0	38	0.0475
ISS	24.8 ± 14.8	72	21.1 ± 14.8	40	0.1927
Survival	9 yes / 63 no	72	8 yes / 32 no	40	0.4100
Surviving vs. Deceased patients					
Length of overall hospitalization	34.2 ± 27.6		12.0 ± 18.9	17	< 0.0001
Haematoma volume (ml)	650.4 ± 413.7	95	1093.3 ± 521.9	17	0.0002
ISS	21.5 ± 13.7	95	34.8 ± 16.1	17	0.0015
Surgical procedures after angiography	1.1 ± 1.1		0.6 ± 1.1	17	0.072
Share of microcoils (% of total)	63.1 ± 46.5	89	28.5 ± 46.8	14	0.0078
PTT (s)	44.7 ± 24.7	95	71.2 ± 53.4	17	0.0114
Hb (g/l)	97.1 ± 26.2	95	77.6 ± 27.5	17	0.0123
Microcoils used	64 yes / 25 no	89	5 yes / 9 no	14	0.0127
Transfusion received (any)	0.8 ± 0.4	95	0.9 ± 0.3	17	0.463
Packed red blood cells	4.5 ± 6.3	95	8.8 ± 2.1	17	0.021
Fresh frozen plasma	0.3 ± 0.8	95	1.0 ± 1.4	17	0.046
Platelet concentrate	2.8 ± 6.4	95	5.5 ± 4.2	17	0.077
Length of microcoils (mm)	427.5 ± 646.0	89	227.9 ± 450.8	14	0.0473
Surgical procedures before angiography	0.1 ± 0.3	95	0.2 ± 0.4	17	0.669
Length of intensive care stay	19.5 ± 21.9	95	18.6 ± 20.2	17	0.741

*Hb* Haemoglobin, *ISS* Injury Severity Score, *PTT* Partial thromboplastin time

### Comparison of older and younger patients

The strong predominance of men among the patients under age 65 (49 men vs. 17 women; *p* = 0.0099) was striking. Younger patients had significantly more severe injuries, more surgical interventions, greater haematoma volume, lower Hb levels and greater need for transfusion than older patients, but the mortality rate was the same in both groups (Table 3).

### Comparison of men and women

Women were significantly older than men (63.9 ± 19.7 years vs. 53.5 ± 19.2 years; *p* = 0.0051). Despite having the same ISS values and pelvic injury patterns according to the classification adopted by the Orthopaedic Trauma Association (OTA), fewer operations were performed on women than on men (Table 3). The time to angiography was significantly longer than in men, and the mortality rate was twice as high as among men (Table 3).

### Comparison between deceased and surviving patients

The deceased patients had significantly greater haematoma volumes than the surviving patients, lower Hb levels, and longer partial thromboplastin times (PTT) (Table 3). Microcoils had been used less often in the deceased group than in survivors. When microcoils were used, their total length was shorter, and the percentage of all coils used was lower than in survivors (Table 3).

### Logistic regression analyses

The decisive factors with respect to mortality were higher ISS before age, haematoma volume, and Hb level (Table 4), while the number of sources of bleeding was eliminated in the stepwise forward analysis. With respect to the need for re-angiography, only the circumstance of an operation performed prior to the initial angiography was relevant (Table 4). Macrocoils tended to be used more when the prothrombin time was short. The more packed red blood cells that were administered and the more macrocoils were used, the fewer microcoils were used. The more vessels that had been injured, the more likely it was that particles were used, but not when surgery had already been performed (Table 4).

**Table 4:** Regression analysis

Logistic Regression analysis	Wald	Odds ratio	Sig.
Dependent: Survival^a^			
Hb (g/l)	3.915	1.028	0.048
Haematoma volume (ml)	4.445	0.999	0.035
Age (years)	6.249	0.941	0.012
ISS	7.361	0.933	0.035
Dependent: Re-do Angiography^a^			
Surgery before angiography	7.508	5.533	0.006
Dependent: Use of Macrocoils^b^			
Hb (g/l)	0.496	0.991	0.481
Crossover technique	0.869	0.515	0.351
Direct or secondary admission	1.179	0.544	0.278
Duration initial CT-angiography (min)	2.036	1.004	0.154
Duration accident-angiography (min)	2.083	0.996	0.149
Age (years)	2.239	1.024	0.135
Arterial calcification	2.868	0.087	0.090
Prothrombine time (%)	2.958	0.978	0.085
Dependent: Use of Microcoils^a^			
Use of macrocoils	7.100	0.251	0.008
Transfusion received	9.519	0.239	0.002
Dependent: Use of Particles^a^			
Surgery before angiography	3.543	0.268	0.060
Number of injured arteries	8.601	2.030	0.003
Linear Regression analysis	B	β	Sig. (t- Test)
Dependent: Duration Trauma to Angiography^c^			
OTA classification	−221.298	−0.189	0.047
Anticoagulation	612.148	0.242	0.011
Surgery before angiography	1066.772	0.336	< 0.001
Dependent: Hematoma^c^			
OTA classification	−115.568	−0.240	0.028
ISS	11.324	0.375	0.001
Dependent: Duration of Angiography and TAE^c^			
Use of particles	−19.256	−0.214	0.024
ISS	−0.639	−0.215	0.019
Number of injured arteries	17.149	0.346	< 0.001
Dependent: Hospitalization^c^			
Duration of angiography	0.108	0.172	0.018
ISS	0.736	0.398	< 0.001
Number of macrocoils used	0.807	0.149	0.039
Crossover technique	17.280	0.216	0.004
Re-do angiography	30.730	0.394	< 0.001
Survival	35.369	0.442	< 0.001

*Hb* Haemoglobin, *ISS* Injury Severity Score, *OTA* Orthopaedic Trauma Association

^a^Forward stepwise method

^b^Enter method

^c^Stepwise method

### Linear regression analyses

Linear regression analyses identified operations prior to embolization and the intake of anticoagulants as factors prolonging the time to angiography and more severe injuries according to OTA as factors reducing the time to angiography. The ISS had a positive effect, but the severity of the pelvic injury according to OTA had a protective effect on the haematoma volume (Table 4). A larger number of injured vessels and a high ISS prolonged the angiography and the use of particles shortened the angiography. The hospital stay was prolonged by re-angiography, a high ISS, long duration of angiography and the number of macrocoils used.

### Propensity score analyses

The propensity score analyses established the following patient-related risk factors for mortality: low haemoglobin associated with a large haematoma volume and high need for transfusion. Procedure-related risk factors were a short length of coils and low total number of coils resulting from a lower number of microcoils used (Table 5). Performing surgical procedures could not be explained by the more severe trauma in patients or by any other factor. Surgery resulted in a pronounced delay of angiography and was associated with a higher volume of haematoma. Re-angiographies were needed in patients with haemorrhage of the parietal branches of the internal iliac artery such as the superior and inferior gluteal artery. Furthermore, particles were used less frequently in these patients. The hospital stay was prolonged dramatically in these patients by more than one month.

**Table 5:** Comparison of propensity-score-matched patient groups

	Deceased patients			Surviving patients				Re-do Angiography			No re-do Angiography				Surgical procedures before Angiography			No surgery before Angiography			
Fisher’s exact test			*n*_1_			*n*_2_	*p* value			*n*_1_			*n*_2_	*p* value			*n*_1_			*n*_2_	*p* value
Gender	7 m	8 f	15	16 m	13 f	29	0.752	11 m	5 f	16	22 m	10 f	32	1.000	11 m	4 f	15	22 m	8 f	30	1.000
Surgical procedures	3 y	12 n	15	1 y	28 n	29	0.107	6 y	10 n	16	5 y	27 n	32	0.144	-	-	-	-	-	-	-
Direct or secondary admission	9 y	6 n	15	15 y	14 n	29	0.752	6 y	10 n	16	12 y	20 n	32	1.000	7 y	8 n	15	20 y	10 n	30	0.218
Transfusion received	13 y	2 n	15	16 y	13 n	29	0.010	11 y	5 n	16	17 y	15 n	32	0.363	11 y	4 n	15	17 y	13 n	30	0.341
Oral anticoagulation	4 y	11 n	15	10 y	19 n	29	0.738	3 y	13 n	16	5 y	27 n	32	1.000	2 y	13 n	15	6 y	24 n	30	0.699
Microcoils used	5 y	10 n	15	26 y	3 n	29	0.000	10 y	6 n	16	19 y	13 n	32	1.000	6 y	9 n	15	18 y	12 n	30	0.226
Macrocoils used	3 y	12 n	15	4 y	25 n	29	0.675	5 y	11 n	16	6 y	26 n	32	0.468	4 y	11 n	15	6 y	24 n	30	0.710
Particles used	8 y	7 n	15	12 y	17 n	29	0.532	3 y	13 n	16	15 y	17 n	32	0.068	3 y	12 n	15	14 y	16 n	30	0.110
Re-do angiography	2 y	13 n	15	6 y	23 n	29	0.695	-	-	-	-	-	-	-	6 y	9 n	15	6 y	24 n	30	0.174
Deceased	-	-	-	-	-	-	-	2 y	14 n	16	5 y	27 n	32	1.000	3 y	12 n	15	5 r	25 l	30	1.000
Mann-Whitney Test	${\overset{-}{x}}_1\pm {\sigma}_1$		*n*_1_	${\overset{-}{x}}_2\pm {\sigma}_2$		*n*_2_	*p* value	${\overset{-}{x}}_1\pm {\sigma}_1$		*n*_1_	${\overset{-}{x}}_2\pm {\sigma}_2$		*n*_2_	*p* value	${\overset{-}{x}}_1\pm {\sigma}_1$			${\overset{-}{x}}_2\pm {\sigma}_2$			*p* value
Patient age (years)	61.8 ± 17.1		15	63.3 ± 19.0		29	0.718	55.8 ± 21.5		16	54.5 ± 21.1		32	0.910	55.9 ± 17.9		15	52.9 ± 20.9		30	0.690
Accident till angiography (min)	287.8 ± 212.4		15	582.6 ± 725.8		29	0.752	976.9 ± 2011.7		16	546.3 ± 1054.3		32	0.774	1374.0 ± 2450.5		15	365.0 ± 447.1		30	0.035
CT till angiography (min)	192.7 ± 216.8		15	422.7 ± 655.9		29	0.194	692.4 ± 2072.1		16	234.6 ± 236.2		32	0.261	853.5 ± 2145.3		15	202.5 ± 273.0		30	0.387
Duration angiography (min)	74.2 ± 51.0		15	89.2 ± 57.4		29	0.561	78.9 ± 44.8		16	76.5 ± 43.9		32	0.935	84.9 ± 56.6		15	71.7 ± 41.9		30	0.428
HB (g/l)	77.0 ±29.3		15	94.6 ± 19.7		29	0.036	82.0 ± 28.5		16	95.9 ± 24.4		32	0.087	89.5 ± 25.1		15	86.4 ± 26.5		30	0.919
PTT (s)	70.7 ± 57.1		15	44.4 ± 13.7		29	0.185	56.2 ± 29.6		16	52.6 ± 43.2		32	0.117	62.5 ± 57.0		15	55.1 ± 37.4		30	0.919
Quick (%)	55.2 ±29.2		15	61.9 ± 26.7		29	0.496	59.1 ± 23.6		16	67.6 ± 26.1		32	0.315	65.3 ± 29.0		15	61.3 ± 24.3		30	0.734
No. of arteries injured	2.4 ± 1.1		15	2.3 ± 1.0		29	0.955	1.8 ± 0.8		16	1.8 ± 1.0		32	0.724	1.8 ± 0.8		15	1.7 ± 0.8		30	0.598
Overall coil length (mm)	393.0 ± 536.6		15	704.3 ±627.6		29	0.035	584.4 ± 794.9		16	352.0 ± 459.4		32	0.220	297.3 ± 463.4		15	485.7 ± 684.6		30	0.147
Overall No. of coils	7.0 ± 7.6		15	12.7 ± 7.8		29	0.017	9.2 ± 8.1		16	6.7 ± 8.1		32	0.220	5.5 ± 8.3		15	6.9 ± 5.8		30	0.120
ISS	35.2 ± 17.1		15	22.8 ± 14.6		29	0.024	28.5 ± 13.7		16	20.8 ± 14.2		32	0.061	26.5 ± 13.4		15	27.7 ± 17.3		30	0.948
OTA classification	2.3 ± 0.8		15	2.3 ± 0.8		29	0.760	2.4 ± 0.7		16	1.9 ± 1.0		32	0.063	2.5 ± 0.9		15	2.1 ± 0.9		30	0.189
Arterial calcification (cm^3^)	0.49 ± 0.89		15	0.39 ± 1.14		29	0.656	0.27 ± 0.43		16	0.28 ± 1.1		32	0.968	0.11 ± 0.18		15	0.19 ± 0.36		30	0.902
Haematoma volume (ml)	1031.3 ± 517.8		15	518.2 ± 258.0		29	0.000	770.9 ± 370.1		16	739.8 ± 454.9		32	0.638	887.9 ± 497.6		15	584.4 ± 334.6		30	0.061
Share of fibered coils (%)	29.4 ± 44.5		15	50.1 ± 43.6		29	0.140	55.8 ± 42.3		16	44.6 ± 48.4		32	0.339	41.3 ± 49.8		15	45.1 ± 47.1		30	0.692
Hospitalization (days)	10.4 ± 17.4		15	35.9 ± 29.6		29	0.000	63.1 ± 31.9		16	19.9 ± 19.0		32	0.000	40.3 ± 28.2		15	26.9 ± 19.7		30	0.181
Overall coil length (mm)	393.0 ± 536.6		15	704.3 ±627.6		29	0.035	584.4 ± 794.9		16	352.0 ± 459.4		32	0.220	297.3 ± 463.4		15	485.7 ± 684.6		30	0.147
Overall No. of coils	7.0 ± 7.6		15	12.7 ± 7.8		29	0.017	9.2 ± 8.1		16	6.7 ± 8.1		32	0.220	5.5 ± 8.3		15	6.9 ± 5.8		30	0.120
No. of microcoils used	4.1 ± 6.9		15	11.2 ± 8.2		29	0.002	5.7 ± 7.3		16	5.8 ± 7.4		32	0.906	2.9 ± 4.8		15	4.9 ± 5.8		30	0.212
No. of macrocoils used	2.9 ± 6.0		15	1.5 ± 8.2		29	0.487	3.5 ± 6.9		16	0.9 ± 2.5		32	0.140	2.5 ± 6.0		15	2.0 ± 4.1		30	0.461

## Discussion

This study showed that the mortality rate depended significantly on a high ISS, age, haematoma volume and Hb level. The propensity score analyses showed that a short total length of coils and the use of too few microcoils were procedure-related factors that were highly significant for mortality rates.

The hospital stay was prolonged by re-angiography, elevated ISS, the duration of the initial angiography and the number of macrocoils used.

If surgical procedures were performed before the angiography, the time until angiography was prolonged by more than 10 h and the haematoma volume increased by more than 200 ml. However, the propensity score analyses did not confirm a more frequent occurrence of re-angiographies when other surgical procedures had been performed prior to the initial angiography. No evidence was found for a possible reason why the initial decision for surgery had been made in the respective cases.

Re-angiographies were performed in patients with haemorrhage in the parietal branches of the internal iliac artery such as the superior and inferior gluteal arteries and who generally had not been treated with particles.

The duration of the initial angiography was shortened when particles were used. Macrocoils were used primarily when the prothrombin time, an indicator for extensive tissue damage, was short. The more macrocoils had been used, the fewer microcoils were used. In women, the time to angiography was longer, fewer operations were performed and the mortality rate was higher than in men.

With respect to possible conclusions from the results, there are some limitations of this study that must be considered. The first limitation is the retrospective study design that may mean that some data from older examinations may be incomplete. For example, the number of packed red blood cell transfusions may have been underestimated. Although the group of 112 patients observed in this study can be considered to be large compared with literature [17, 19], the possibilities of multivariate analyses that can be conducted in it are nevertheless limited. For example, no analyses of the cause of the accident or the injured vessels could be made. It must also be taken into account that in retrospective studies, effects of factors on other parameters must be assessed with caution, as there may be a bias due to factors that may not have been documented and are therefore unknown. For example, if a patient had been treated with pelvic packing instead of angiography in the observation period, there would have been a selection bias whose effect on the results of the study could no longer be determined. Since it cannot be ruled out that, for example, a causal factor that was ultimately responsible for affecting a parameter was not included in the data and analysis, it is possible that some of the factors indentified as causal in the regression models are actually only covariables. Of course, it is directly evident and intuitively true in the sense of René Descartes’ “*Discourse of the Method*” that an operation needs time and can thus be considered to be a causal factor for a delay until angiography — however, that does not explain the question of whether an angiography would even have been performed without the operation or the patient would perhaps have died.

Finally, the study impact might be limited due to lack of a control group and the fact that the use of pelvic binders could not be evaluated accurately. Pelvic binders are applied routinely for initial pelvic stabilisation in our institution, but neither their application to the patient nor their removal had been documented sufficiently. They may be a strong predictor for outcome, but this has not been analysed.

The predominance of OTA type B and C injuries within the cohort could be explained by the fact that severe haemorrhage occurs less frequently with less severe injuries of the bony pelvis [19, 27]. Since at least 8.9% of patients had severe haemorrhage without bone injuries, further imaging should also be carried out in those cases. The location of the bleeding, usually the territory of the internal iliac artery, corresponds well with the literature [2, 6]. The close proximity of parietal branches to the bony structures of the pelvis could cause shearing and bruising of the vessels on the bone and cuts from bone fragments if a fracture occurred. If these parietal branches are affected by haemorrhage, it is presumably therefore more probable that a re-angiography will be needed because these vessels are especially well collateralised and the haemorrhage possibly does not stem from only one side.

The characteristic age distribution can be attributed to the different origins of trauma in different phases of life. While in younger patients, risky behaviour in sport, recreation and traffic is assumed, pre-existing osteoporosis in elderly patients could make them prone to fractures after what are usually simple falls [1, 3]. The lower ISS and the less frequent surgeries in older patients in comparison with younger ones are indications of the low-energy trauma in older patients [1]. Accordingly, the haematoma volume was smaller in the older patients. The shorter time between the trauma and the start of angiography in patients below age 65 could be related to the more extensive injuries that are likely to be associated with faster admission to the hospital and more rapid initiation of treatment. The significantly longer duration of the angiography in elderly patients can be interpreted to mean that they could initially be more stable because of the smaller extent of haemorrhage and therefore less urgency is perceived. This is consistent with the use of a significantly higher number of microcoils, whose application is somewhat more time consuming than other coils. Their advantage is the possibility of probing smaller branches more selectively using microcatheters and then selectively embolizing them. The higher mortality rate in elderly patients that is consistent with literature [28] can possibly be attributed to their lower physiological reserves on the one hand and to more frequent comorbidities on the other hand [3, 4]. The significantly higher age of women compared to men at the time of the accident, which is also consistent with literature [29], could explain the higher mortality rate of women [4]. However, there is no explanation for the fact that significantly fewer operations were performed than in men for injuries of the same severity and that the time until angiography was longer than for men except that women were disadvantaged with respect to treatment.

The deceased patients had significantly lower Hb levels and accordingly were given more packed red blood cell transfusions. Both factors are known to be associated with a higher mortality rate [30, 31]. The ISS and the volume of the haematoma were greater in the deceased. The prolonged PTT could suggest disseminated intravascular coagulation. All these factors can be attributed to the severity of the injury – indirectly in the case of ISS, Hb level, and the volume of the haematoma and directly for packed red blood cell administration. Theoretically, the only way to affect these factors at least in part is to achieve effective haemostasis as quickly as possible and thus perform an angiography as soon as possible. However, the propensity score analyses showed two new, previously unknown, major risk factors for mortality: a considerably shorter total coil length and the much less frequent use of microcoils. This suggests that in an angiography of the pelvic vessels after a trauma, particular importance should be given to thorough peripheral embolization with a sufficient number of microcoils, especially for haemorrhage from the parietal branches of the internal iliac artery. Contrary to the widespread opinion of many interventional radiologists, it should not be relied on that haemorrhage that is reduced by the application of a few coils will resolve over time due to coil-induced thrombosis of the vessel. On the contrary, thorough embolization should continue until complete stasis, especially as the ability of blood to coagulate is reduced by the trauma itself and by blood loss. This hypothesis is supported by the fact that the main cause of death was exsanguination due to severe trauma, before multi organ failure and head injuries. By means of modern angioembolization arterial haemorrhage can be controlled sufficiently and effectively but neither can venous haemorrhage nor bleedings from bone fractures be treated. Therefore, the application of pelvic packing [15] after arterial angioembolization has to be considered as an hybrid approach in situations where the patients remain unstable after embolization, i.e. when the Hb level is continuously falling. Some arteries might be difficult to reach surgically [15] and opening of the retroperitoneal space in presence of arterial bleeding may even pose a further risk for the patient. Due to our finding that surgery before angioembolization was a strong predictor for redo-angiography, we suggests to perform angiography first. However, prospective trials evaluating best practise whether to perform pelvic packing or angioembolization first are presently not available. The mortality of 15.2% in the this study is very low compared with the literature [10, 22, 32–35] suggesting a mortality of at least 40% - 60% – especially considering the number of patients suffering from major trauma in the our study. The fatal outcome of the 10 patients deceased due to exsanguination might could have been avoided by choosing a hybrid approach of packing and embolization. There is an urgent need for prospective studies aiming to further improve patient selection and management for the procedures.

Patients who later underwent re-angiography were initially transferred from other hospitals to the first level trauma centre more often than others. Surgical procedures before the initial angiography were markedly more frequent in this group. The haematoma volumes in these patients were somewhat greater, the Hb levels lower, and the partial thromboplastin times prolonged. Since the severity of the injuries did not differ from those of patients who underwent only one angiography, it is possible that the longer waiting period led to greater blood loss and consumption of coagulation factors, as suggested by the propensity score analyses. Since the injuries were not more severe than in other patients, it can also be assumed that the operations performed before the angiographies were given priority more due to an individual decision than to vital indications. All patients with active arterial bleeding after a blunt pelvic trauma should therefore initially undergo an angiography. Since fewer particles were used in the initial angiography of patients who underwent multiple angiographies than in patients who did not undergo a re-angiography, particles should be used for every pelvic haemorrhage unless contraindicated.

Since the length of the hospital stay in the multivariate analyses proved to be dependent on the re- angiographies, an higher ISS, the duration of the initial angiography and the number of macrocoils used, a reduction of the re-angiography rate might also contribute to a reduction in the length of stay. However, the ISS itself cannot be affected, nor its impact on the duration of the initial angiography or the number of bleeding sites and thus the vessels to be probed. However, the propensity score analyses showed that trauma to the parietal branches of the internal iliac artery was an additional risk factor for re-angiography. If these branches are affected, particular attention should be given to thorough peripheral embolization with the application of particles as far peripheral as possible. Moreover, the use of particles is the only factor for reducing the duration of the initial angiography that can be modified. The number of the macrocoils used must be understood in light of achieving rapid haemostasis through the fastest possible embolization of injured major vessels without the highly selective probing of other branches and therefore can also not be influenced.

## Conclusion

In summary, older patients had mainly “low-energy” traumas while severe polytraumas were frequently observed among younger people. The severity of the injury, the haematoma volume, low Hb levels and advanced age were relevant for survival. However, the length of coils and the use of microcoils were also found to be factors that could be modified. Embolization should therefore always be carried out as far peripheral as possible. As many microcoils as are needed until complete stasis is achieved should be used.

Since angiographies were delayed especially in women and older patients, the outcomes for these groups might be significantly improved if they received equal treatment. The initial use of microparticles in addition to coils may reduce both the duration of the angiography and the re-angiography rate. Macrocoils are the treatment of choice for severe bleeding from large vessels, but in general, if there is time, preference should be given to microcoils in highly selective vessels to the bleeding source combined with particles.

## Abbreviations

A&E: Accidence and Emergency
AIS: Abrreviated Injury Scale
CT: Computed Tomography
Hb: Heamoglobin
ISS: Injury Severity Score
kV: Kilo-Volt
OTA: Orthopaedic Trauma Association
PTT: Partial Thromboplastin Times
PVA: Polyvenyl Alcohol Particles
RIS: Radiology Information System

## References

[1] Krappinger D, Kammerlander C, Hak DJ, Blauth M (2010). Low-energy osteoporotic pelvic fractures. Arch Orthop Trauma Surg.

[2] Krappinger D, Zegg M, Jeske C, El Attal R, Blauth M, Rieger M (2012). Hemorrhage after low-energy pelvic trauma. The journal of trauma and acute care surgery.

[3] Alost T, Waldrop RD (1997). Profile of geriatric pelvic fractures presenting to the emergency department. Am J Emerg Med.

[4] Henry SM, Pollak AN, Jones AL, Boswell S, Scalea TM (2002). Pelvic fracture in geriatric patients: a distinct clinical entity. J Trauma.

[5] Ben-Menachem Y, Coldwell DM, Young JW, Burgess AR (1991). Hemorrhage associated with pelvic fractures: causes, diagnosis, and emergent management. AJR Am J Roentgenol.

[6] Jeske HC, Larndorfer R, Krappinger D, Attal R, Klingensmith M, Lottersberger C, Dunser MW, Blauth M, Falle ST, Dallapozza C (2010). Management of hemorrhage in severe pelvic injuries. J Trauma.

[7] White CE, Hsu JR, Holcomb JB (2009). Haemodynamically unstable pelvic fractures. Injury.

[8] Osborn PM, Smith WR, Moore EE, Cothren CC, Morgan SJ, Williams AE, Stahel PF (2009). Direct retroperitoneal pelvic packing versus pelvic angiography: a comparison of two management protocols for haemodynamically unstable pelvic fractures. Injury.

[9] Stephen DJ, Kreder HJ, Day AC, McKee MD, Schemitsch EH, ElMaraghy A, Hamilton P, McLellan B (1999). Early detection of arterial bleeding in acute pelvic trauma. J Trauma.

[10] Agolini SF, Shah K, Jaffe J, Newcomb J, Rhodes M, Reed JF (1997). Arterial embolization is a rapid and effective technique for controlling pelvic fracture hemorrhage. J Trauma.

[11] Slater SJ, Barron DA (2010). Pelvic fractures-a guide to classification and management. Eur J Radiol.

[12] Theumann NH, Verdon JP, Mouhsine E, Denys A, Schnyder P, Portier F (2002). Traumatic injuries: imaging of pelvic fractures. Eur Radiol.

[13] Pizanis A, Pohlemann T, Burkhardt M, Aghayev E, Holstein JH (2013). Emergency stabilization of the pelvic ring: clinical comparison between three different techniques. Injury.

[14] Ball CG (2014). Damage control resuscitation: history, theory and technique. Canadian journal of surgery Journal canadien de chirurgie.

[15] Suzuki T, Smith WR, Moore EE (2009). Pelvic packing or angiography: competitive or complementary?. Injury.

[16] Hawkins L, Pomerantz M, Eiseman B (1970). Laparotomy at the time of pelvic fracture. J Trauma.

[17] Papakostidis C, Kanakaris N, Dimitriou R, Giannoudis PV (2012). The role of arterial embolization in controlling pelvic fracture haemorrhage: a systematic review of the literature. Eur J Radiol.

[18] Frevert S, Dahl B, Lonn L (2008). Update on the roles of angiography and embolization in pelvic fracture. Injury.

[19] El-Haj M, Bloom A, Mosheiff R, Liebergall M, Weil YA (2013). Outcome of angiographic embolization for unstable pelvic ring injuries: factors predicting success. Injury.

[20] Lubarsky M, Ray CE, Funaki B (2009). Embolization agents-which one should be used when? Part 1: large-vessel embolization. Semin Interv Radiol.

[21] Lubarsky M, Ray C, Funaki B (2010). Embolization agents-which one should be used when? Part 2: small-vessel embolization. Semin Interv Radiol.

[22] Matalon TS, Athanasoulis CA, Margolies MN, Waltman AC, Novelline RA, Greenfield AJ, Miller SE (1979). Hemorrhage with pelvic fractures: efficacy of transcatheter embolization. AJR Am J Roentgenol.

[23] White JB, Ken CG, Cloft HJ, Kallmes DF (2008). Coils in a nutshell: a review of coil physical properties. AJNR Am J Neuroradiol.

[24] Osuga K, White RI (2003). Micronester: a new pushable fibered microcoil for embolotherapy. Cardiovasc Intervent Radiol.

[25] Rosenbaum PR, Rubin DB (1985). Constructing a control group using multivariate matched sampling methods that incorporate the propensity score. Am Stat.

[26] Iacus SM, King G, Porro G (2011). Multivariate matching methods that are monotonic imbalance bounding. J Am Stat Assoc.

[27] Dyer GS, Vrahas MS (2006). Review of the pathophysiology and acute management of haemorrhage in pelvic fracture. Injury.

[28] Yoshihara H, Yoneoka D (2014). Demographic epidemiology of unstable pelvic fracture in the United States from 2000 to 2009: trends and in-hospital mortality. The journal of trauma and acute care surgery.

[29] Kannus P, Palvanen M, Niemi S, Parkkari J, Jarvinen M (2000). Epidemiology of osteoporotic pelvic fractures in elderly people in Finland: sharp increase in 1970-1997 and alarming projections for the new millennium. Osteoporosis international : a journal established as result of cooperation between the European Foundation for Osteoporosis and the National Osteoporosis Foundation of the USA.

[30] Wong YC, Wang LJ, Ng CJ, Tseng IC, See LC (2000). Mortality after successful transcatheter arterial embolization in patients with unstable pelvic fractures: rate of blood transfusion as a predictive factor. J Trauma.

[31] Knottenbelt JD (1991). Low initial hemoglobin levels in trauma patients: an important indicator of ongoing hemorrhage. J Trauma.

[32] Cook RE, Keating JF, Gillespie I (2002). The role of angiography in the management of haemorrhage from major fractures of the pelvis. J Bone Joint Surg (Br).

[33] Dente CJ, Feliciano DV, Rozycki GS, Wyrzykowski AD, Nicholas JM, Salomone JP, Ingram WL (2005). The outcome of open pelvic fractures in the modern era. Am J Surg.

[34] Hamill J, Holden A, Paice R, Civil I (2000). Pelvic fracture pattern predicts pelvic arterial haemorrhage. Aust N Z J Surg.

[35] Miller PR, Moore PS, Mansell E, Meredith JW, Chang MC (2003). External fixation or arteriogram in bleeding pelvic fracture: initial therapy guided by markers of arterial hemorrhage. J Trauma.

